# Ofatumumab Desensitization Protocol: A Case of Refractory Immune Thrombocytopenic Purpura

**DOI:** 10.7759/cureus.46278

**Published:** 2023-09-30

**Authors:** Audra L Cochran, Meredith M Schuldt, James M Quinn

**Affiliations:** 1 Internal Medicine, Keesler Medical Center, Biloxi, USA; 2 Allergy and Immunology, Wilford Hall Ambulatory Surgical Center, Lackland Air Force Base (AFB), USA

**Keywords:** anti cd-20, biologic treatment, drug-related adverse reactions, refractory itp, desensitization treatment, itp in adult, immune mediated thrombocytopenic purpura (itp), ofatumumab

## Abstract

Chronic immune thrombocytopenic purpura (ITP) is an acquired hematologic condition that involves immune-mediated platelet destruction with resultant bleeding of variable severity. Refractory ITP occurs when patients fail to tolerate and/or respond to multiple treatment modalities. In this case, we examine the clinical course of a 39-year-old female with refractory ITP and discuss how we navigated a multitude of challenges by adapting an established desensitization protocol to meet our patient’s needs. To our knowledge, we describe the first successful desensitization to ofatumumab for use in ITP in the current literature.

## Introduction

Immune thrombocytopenic purpura (ITP) is an autoimmune disorder that involves antibody-mediated destruction of platelets. Complications of ITP include fatigue, minor bleeding (petechiae, purpura, and mucosal bleeding), and even major bleeding such as intracerebral hemorrhage (ICH) [1]. Initial therapy for ITP is the prompt administration of IV corticosteroids, followed by consideration of IV immunoglobulin (IVIG) or Rho(D) immunoglobulin (anti-RhD) for steroid-resistant cases. For patients dependent upon steroids or who are unresponsive to steroids after three to 12 months, second-line therapies include thrombopoietin receptor agonists (TPO-RA) such as romiplostim and anti-CD20 monoclonal antibodies (such as rituximab) per the 2019 American Society of Hematology guidelines. For patients with ITP lasting >12 months despite treatment, splenectomy should be considered as an additional second-line therapy. Further therapies include fostamatinib, an oral tyrosine kinase inhibitor (TKI) of splenic macrophages [2].

We present the case of a complex adult patient with severe chronic refractory ITP who failed traditional treatment with steroids and IVIG as well as multiple second-line therapies. Although newer generations of humanized (obinutuzumab, ocrelizumab, and veltuzumab) and fully human (ofatumumab) anti-CD20 monoclonal antibodies have been developed, there is currently a paucity of data available regarding the use of these new anti-CD20 agents in ITP [3]. In our unique case, we modified an established desensitization protocol for rituximab from Yang et al. into a customized desensitization protocol for ofatumumab with successful remission of ITP [4]. Multiple alterations were made to our protocol to accommodate challenges we encountered, including package insert infusion rate parameters and a prolonged interval between two of the infusions. We describe our solutions to these issues and also display the evolution of our protocol in a series of tables with the hope of helping other physicians who find themselves navigating similar challenges.

## Case presentation

Our patient was a 39-year-old female with Niemann-Pick disease type B (NPD-B), multiple sclerosis (MS), and severe refractory ITP who initially presented with significant thrombocytopenia. The initial platelet count was 35,000/mcL and rapidly reached a nadir of 10,000/mcL. No bleeding complications occurred. After failing treatment with dexamethasone, IVIG, romiplostim (TPO-RA), and fostamatinib (TKI), she was subsequently trialed on multiple anti-CD20 monoclonal therapies. The patient developed serum sickness in association with rituximab on her ninth infusion. Her reaction was characterized by fever, pruritus, paresthesia of the lips, a macular rash evolving into urticaria on her torso and upper extremities, and limited mobility due to prolonged debilitating joint swelling and pain that persisted despite a prolonged steroid course for more than one week after administration of rituximab.

She was then switched to ofatumumab and tolerated the first eight infusions well, but after an extended dosing interval, she developed facial angioedema and generalized urticaria during her ninth ofatumumab infusion. The rate of ofatumumab infusion was slowed, and the patient was premedicated with steroids and antihistamines accordingly, but she developed a similar reaction again. She was then trialed on obinutuzumab and unfortunately experienced another similar adverse reaction with urticaria and angioedema despite premedication. Next, the patient was trialed on fostamatinib (TKI) for nearly a year; however, this medication was ultimately discontinued due to a lack of demonstrated benefit. Since the patient was deemed ineligible for splenectomy due to her underlying NPD-B, the allergy service was consulted for desensitization in the setting of limited treatment options.

Since the patient had no history of serum sickness, vasculitis, or a severe cutaneous adverse reaction (SCAR) such as toxic epidermal necrolysis (TEN) or Stevens-Johnson syndrome (SJS) to either ofatumumab or obinutuzumab, temporary induction of tolerance to either one of these medications was felt to have the best risk/benefit profile. After shared decision-making with the patient and hematology, ofatumumab with a loading dose of 300mg and a subsequent 1000mg treatment dose was selected for the patient’s refractory ITP.

Our patient was pretreated with methylprednisolone 50mg IV x1, cetirizine 10mg PO x1, famotidine 20mg PO x1, and montelukast 10mg PO x1, administered one to two hours prior to each infusion. Normal saline was also infused at 150cc to 250cc/hr during each infusion, with instructions to increase the rate to 500cc/hr should a reaction occur. As needed, medications were kept on hand, including an epinephrine autoinjector 0.3mg intramuscular (IM) as needed for anaphylaxis, ondansetron 4mg to 8mg IV as needed for nausea, lorazepam 0.5mg to 1.0mg IV as needed for anxiety, albuterol 90mcg inhaled as needed for bronchospasm, and H1/H2 blockade with diphenhydramine 25mg PO and famotidine 20mg PO as needed for hives or angioedema. No adverse reactions occurred during any ofatumumab desensitization infusions.

The initial desensitization with ofatumumab occurred with a 4-bag, 16-step protocol for a total dose of 300mg over 6.63 hours (Table 1). Protocol alteration was made one week later to adhere to package insert infusion rate parameters with escalation to a higher total dose of 1000mg over 7.65 hours. Additionally, since there was a nationwide shortage of ofatumumab, each bag of solution was mixed to a slightly higher concentration to minimize medication waste (Table 2). The patient required a third desensitization with ofatumumab (Table 3) since her diagnosis of COVID-19 led to a prolonged interval between infusions (two months instead of one month). Our plan to provide monthly ofatumumab infusions over the next year was projected to place a significant demand on both the patient’s time and our infusion clinic’s resources, so the protocol was tailored once again to reduce total infusion time. The result was a 3-bag, 14-step protocol that was similarly well-tolerated by the patient and was infused over 5.57 hours (Table 4). The patient continued to tolerate monthly infusions of ofatumumab 1000mg and exhibited improvement in her ITP with normalization of her platelet counts.

**Table 1:** Ofatumumab desensitization round 1

Step	Bag of Solution	Concentration (mg/mL)	Rate (mL/hr 0	Infusion Time (in minutes)	Volume (mL)	Dose of Step (mg)	Cumulative Dose (mg)
1	1st	0.00075	2.5	15	0.625	0.000469	0.000469
2	1st	0.00075	5.0	15	1.250	0.000938	0.001407
3	1st	0.00075	10.0	15	2.500	0.001875	0.003282
4	1st	0.00075	20.0	15	5.000	0.003750	0.007032
5	2nd	0.01200	2.5	15	0.625	0.007500	0.014532
6	2nd	0.01200	5.0	15	1.250	0.015000	0.029532
7	2nd	0.01200	10.0	15	2.500	0.030000	0.059532
8	2nd	0.01200	20.0	15	5.000	0.060000	0.119532
9	3rd	0.12000	5.0	15	1.250	0.150000	0.269532
10	3rd	0.12000	10.0	15	2.500	0.300000	0.569532
11	3rd	0.12000	20.0	15	5.000	0.600000	1.169532
12	3rd	0.12000	40.0	15	10.000	1.200000	2.369532
13	4th	1.20000	10.0	15	2.500	3.000000	5.369532
14	4th	1.20000	20.0	15	5.000	6.000000	11.369532
15	4th	1.20000	40.0	15	10.000	12.000000	23.369532
16	4th	1.20000	80.0	173	230.667	276.800000	300.169532

**Table 2:** Ofatumumab desensitization round 2

Step	Bag of Solution	Concentration (mg/mL)	Rate (mL/hr)	Infusion Time (in minutes)	Volume (mL)	Dose of Step (mg)	Cumulative Dose (mg)
1	1st	0.00100	2.5	15	0.625	0.000625	0.000625
2	1st	0.00100	5.0	15	1.250	0.001250	0.001875
3	1st	0.00100	10.0	15	2.500	0.002500	0.004375
4	1st	0.00100	20.0	15	5.000	0.005000	0.009375
5	2nd	0.02000	2.5	15	0.625	0.012500	0.021875
6	2nd	0.02000	5.0	15	1.250	0.025000	0.046875
7	2nd	0.02000	10.0	15	2.500	0.050000	0.096875
8	2nd	0.02000	20.0	15	5.000	0.100000	0.196875
9	3rd	0.20000	5.0	15	1.250	0.250000	0.446875
10	3rd	0.20000	10.0	15	2.500	0.500000	0.946875
11	3rd	0.20000	20.0	15	5.000	1.000000	1.946875
12	3rd	0.20000	40.0	15	10.000	2.000000	3.946875
13	4th	2.00000	10.0	30	5.000	10.000000	13.946875
14	4th	2.00000	20.0	30	10.000	20.000000	33.946875
15	4th	2.00000	40.0	30	20.000	40.000000	73.946875
16	4th	2.00000	80.0	30	40.000	80.000000	153.946875
17	4th	2.00000	160.0	159	424.000	848.000000	1001.946875

**Table 3:** Ofatumumab desensitization round 3

Step	Bag of Solution	Concentration (mg/mL)	Rate (mL/hr)	Infusion Time (in minutes)	Volume (mL)	Dose of Step (mg)	Cumulative Dose (mg)
1	1st	0.00250	2.5	15	0.625	0.001563	0.001563
2	1st	0.00250	5.0	15	1.250	0.003125	0.004688
3	1st	0.00250	10.0	15	2.500	0.006250	0.010938
4	1st	0.00250	20.0	15	5.000	0.012500	0.023438
5	2nd	0.04000	2.5	15	0.625	0.025000	0.048438
6	2nd	0.04000	5.0	15	1.250	0.050000	0.098438
7	2nd	0.04000	10.0	15	2.500	0.100000	0.198438
8	2nd	0.04000	20.0	15	5.000	0.200000	0.398438
9	3rd	0.40000	5.0	15	1.250	0.500000	0.898438
10	3rd	0.40000	10.0	15	2.500	1.000000	1.898438
11	3rd	0.40000	20.0	15	5.000	2.000000	3.898438
12	3rd	0.40000	40.0	15	10.000	4.000000	7.898438
13	4th	2.00000	10.0	30	5.000	10.000000	17.898438
14	4th	2.00000	20.0	30	10.000	20.000000	37.898438
15	4th	2.00000	40.0	30	20.000	40.000000	77.898438
16	4th	2.00000	80.0	30	40.000	80.000000	157.898438
17	4th	2.00000	160.0	30	80.000	160.000000	317.898438
18	4th	2.00000	320.0	64	341.333	682.666667	1000.565105

**Table 4:** Ofatumumab desensitization round 4

Step	Bag of Solution	Concentration (mg/mL)	Rate (mL/hr)	Infusion Time (in minutes)	Volume (mL)	Dose of Step (mg)	Cumulative Dose (mg)
1	1st	0.02000	2.5	15	0.625	0.012500	0.012500
2	1st	0.02000	5.0	15	1.250	0.025000	0.037500
3	1st	0.02000	10.0	15	2.500	0.050000	0.087500
4	1st	0.02000	20.0	15	5.000	0.100000	0.187500
5	2nd	0.20000	5.0	15	1.250	0.250000	0.437500
6	2nd	0.20000	10.0	15	2.500	0.500000	0.937500
7	2nd	0.20000	20.0	15	5.000	1.000000	1.937500
8	2nd	0.20000	40.0	15	10.000	2.000000	3.937500
9	3rd	2.00000	10.0	30	5.000	10.000000	13.937500
10	3rd	2.00000	20.0	30	10.000	20.000000	33.937500
11	3rd	2.00000	40.0	30	20.000	40.000000	73.937500
12	3rd	2.00000	80.0	30	40.000	80.000000	153.937500
13	3rd	2.00000	160.0	30	80.000	160.000000	313.937500
14	3rd	2.00000	320.0	64	341.333	682.666667	996.604167

## Discussion

Adverse drug reactions (ADRs) can be broken down into two major types: type A reactions, which account for approximately 90% of ADRs and are mediated by the drug itself (commonly referred to as side effects); and type B reactions, which are drug hypersensitivity reactions (DHRs) mediated by the immune system’s overstated response to the drug [5]. Type B drug hypersensitivity reactions can be broken down into seven subtypes: (1) type 1 immediate reactions (both IgE-mediated and non-IgE-mediated); (2) cytokine release; (3) mixed; (4) type 2 reactions (cytotoxic); (5) type 3 reactions (mediated by complement proteins); (6) type 4 delayed reactions (mediated by T-cells); and (7) infusion-related reactions [6]. These subtypes are classified based on their phenotype (clinical presentation), endotype (pathophysiology), and biomarkers (such as tryptase and IL-6) [7]. Our patient’s reaction to ofatumumab was most characteristic of a type I IgE-mediated hypersensitivity reaction, given her immediate reaction phenotype and positive response to antihistamines.

Drug-related hypersensitivity reactions have been occurring more frequently in the last two decades, likely due to the increased development of novel therapeutic agents and increased exposure to chemotherapy, antibiotics, and biologics [6]. Biologics in particular are notorious for a high rate of DHRs, with up to 25% of patients on biologics experiencing a hypersensitivity reaction [7]. Even the first dose of a biologic can potentially lead to a DHR [7], as seen with obinutuzumab in the case of our patient. Hypersensitivity reactions to biologics have been increasing in frequency over time, likely due in part to their growing therapeutic use [8]. Since the first biologic medication was approved by the FDA in 1986, the indications for biologic therapies have expanded exponentially [9]. The clinical utility of biologics now spans a broad spectrum of medical specialties, from hematology and oncology to rheumatology, pulmonology, dermatology, and allergy [8].

Biologics are defined as biotechnological substances, typically proteins or polypeptides, that are either produced from living organisms or manufactured from a product of a living organism [8]. The most common types of biologics are monoclonal antibodies (mAb), fusion proteins, and cytokines [10]. Inherent differences between biologics and other pharmaceutical drugs include their size and how they are ultimately processed. While most drugs are chemicals composed of small molecules, most biologics are large, complex proteins with tertiary polypeptide structures. Biologics are also broken down into peptides in the body like any protein, whereas other drugs undergo renal or hepatic metabolism [9]. Unlike other drugs that sometimes require metabolic activation after administration, biologics do not contain prodrugs [10]. The larger size and more complex structure of mAB increase their potential for immunogenicity, particularly if those mAbs are chimeric or murine in composition [7]. Biologics account for the vast majority of drug desensitization procedures, possibly as high as 72% to 87% of all rapid drug desensitization (RDD) procedures performed [11].

Rapid drug desensitization is indicated for the management of type I hypersensitivity reactions, cytokine release reactions, mixed reactions, and even some type IV hypersensitivity reactions (with the notable exception of SCARs) when the culprit medication is medically necessary with no equally effective alternatives [6]. Risk stratification is performed first and foremost, and patients should be screened for high-risk features such as pregnancy, severe comorbid diseases, severe index DHR, and use of medications such as beta-blockers or angiotensin-converting enzyme inhibitors; while none of these factors are contraindications to RDD, they can increase the risk of breakthrough reactions during a desensitization procedure [12].

Drug desensitization has been found to be safe and effective in multiple studies, with only about 90% of patients having either no reactions at all or only mild reactions [12]. The process of RDD involves gradually administering small doses of a drug and increasing the dose as tolerated over time, with many published dosing regimens available [4]. While drug desensitization is not permanent, the process does induce a temporary tolerance that must be maintained with regular dosing intervals. If the drug is completely cleared from the system before the next exposure, repeat RDD would be required [12]. For instance, our patient required additional desensitization with ofatumumab due to a prolonged interval between infusions when she was diagnosed with COVID-19.

Protocols for RDD may need to be revised to account for breakthrough reactions or unforeseen circumstances. In our patient, a cautious 16-step desensitization plan was initially implemented based upon previously published dosing regimens for similar biologics [4] as a framework; however, there were required alterations to adapt to ofatumumab’s specific dosing intervals. Infusion rates as well as known limitations in supply required additional steps to reduce waste but provide adequate dosing. In the interest of both the patient’s time and clinic resource constraints, the total protocol duration was shortened in its final iteration. The multiple adjustments to our patient’s protocol were made possible due to a multidisciplinary approach.

The success of RDD is dependent on the joint involvement of the primary team, pharmacy, and other specialists [7], and teams that consult allergists have been shown to yield better patient outcomes [11]. Unfortunately, referrals to allergy departments have been estimated to be as low as 4% [11]. In the absence of expert supervision, many patients with DHRs could be placed at risk for future reactions or inappropriately switched to second-line treatments. For this reason, it is recommended that RDD be undertaken by allergists rather than non-experts to optimize outcomes [11].

## Conclusions

Rapid drug desensitization allows allergic patients who would otherwise be subjected to suboptimal treatments to instead receive a wider range of therapeutic options, thereby decreasing their disease burden and increasing their quality of life. Our patient’s positive response to treatment with ofatumumab supports the need for further studies into the use of new anti-CD20 agents in ITP. Finally, this case highlights an initial conservative desensitization protocol that can be customized to meet treatment goals and drug-specific requirements once a patient has been successfully desensitized.
